# Interaction effects of the COVID-19 pandemic and regional deprivation on self-rated health: a cross-sectional study

**DOI:** 10.1186/s12889-024-19814-x

**Published:** 2024-09-02

**Authors:** Hajae Jeon, Junbok Lee, Mingee Choi, Bomgyeol Kim, Sang Gyu Lee, Jaeyong Shin

**Affiliations:** 1 Department of Public Health, Graduate School, Yonsei University, Seoul, Republic of Korea; 2 Health-IT Center, Yonsei University Health System Seoul, Seoul, Republic of Korea; 3 Department of Preventive Medicine, Institute of Health Services Research, Yonsei University College of Medicine, 50 Yonsei-ro, Seodaemun-gu, Seoul, 03722 Republic of Korea

**Keywords:** COVID-19, Socioeconomic factors, Health status indicators, Diagnostic self-evaluation

## Abstract

**Background:**

Recent studies have attempted to analyze the changes in self-rated health (SRH) during the coronavirus disease 2019 (COVID-19) pandemic. However, the results have been inconsistent. Notably, SRH is subjective, and responses may vary across and within countries because of sociocultural differences. Thus, we aimed to examine whether the interaction effects between the COVID-19 pandemic and regional deprivation influenced SRH in South Korea.

**Methods:**

The study population comprised 877,778 participants from the Korea Community Health Survey. The data were collected from 2018 to 2021. Multiple regression analysis was employed to determine the relationship between SRH and the interaction between the COVID-19 pandemic status and the socioeconomic level of residential areas.

**Results:**

The post-pandemic groups (odds ratio [OR] = 2.25, *P* < .0001; OR = 2.29, *P* < .0001) had significantly higher odds of reporting favorable SRH than the pre-pandemic groups (OR = 0.96, *P* < .0001). However, the difference in ORs based on regional socioeconomic status was small.

**Conclusions:**

SRH showed an overall increase in the post-pandemic groups relative to that in the disadvantaged pre-pandemic group. Possible reasons include changes in individuals’ health perceptions through social comparison and the effective implementation of COVID-19 containment measures in South Korea. This paradoxical phenomenon has been named the “Eye of the Hurricane,” as the vast majority of people who had not been infected by the virus may have viewed their health situation more favorably than they ordinarily would.

**Supplementary Information:**

The online version contains supplementary material available at 10.1186/s12889-024-19814-x.

## Background

The coronavirus disease 2019 (COVID-19) pandemic considerably affected daily life [1–4]. To prevent the spread of COVID-19, various measures such as social distancing, telecommuting, and restrictions on private gatherings were implemented, leading to social disruption and isolation. In South Korea, this response included strict compliance with social distancing guidelines with concomitant extensive testing, contact tracing, and isolation of confirmed cases [5, 6] (see Appendix 1). The COVID-19 outbreak and the consequent disruption of daily life generated stress and adversely affected the mental and physical well-being of individuals by reducing social contact [3, 4]. Therefore, investigating the effect of the COVID-19 pandemic on health-related indicators will provide important insights into relevant health measures to improve health.

Self-rated health (SRH) is the most common health measure used in large population surveys. SRH primarily captures individuals’ subjective assessments of their health, although it is also linked to objective health conditions. SRH is an important indicator because it is widely used to examine patterns and disparities in population health in relation to socioeconomic factors [7, 8]. SRH is influenced by a range of complex factors, encompassing the physical characteristics of the shared environment, including walkability, accessibility of public transportation, and availability of healthcare services; socioeconomic and sociocultural characteristics of the local community, and biological and genetic traits of the individuals [9–11].

In a previous study, Tak explored the correlation between regional deprivation levels and the SRH of residents in South Korea while considering the moderating effect of neighborhood relationships [12]. The study revealed notable disparities in health outcomes across various regions. Studies have also analyzed the relationship between physical activity and SRH to understand the changes in health levels resulting from lifestyle modifications and the decline in quality of life during the COVID-19 pandemic [13]. However, these studies primarily focused on lifestyle modifications and did not specifically investigate the differences between the pre- and post-pandemic periods, indicating a limitation in their scope.

Conversely, studies conducted abroad have shown that SRH, which is an integrated evaluation of one’s physical, mental, social, and functional health, tended to improve following the COVID-19 pandemic, despite the pandemic’s negative impact on mental and social health [7, 14, 15]. Notably, SRH is subjective, and responses may vary across and within countries because of sociocultural differences [16]. The impact on the health and well-being of populations is anticipated to differ across countries because of variations in COVID-19 prevalence and regulations as well as pre-existing disparities in well-being and healthcare systems prior to the onset of the pandemic [7, 17]. Therefore, gaps exist in the current literature, and they highlight the need to investigate and analyze the socioeconomic factors and SRH in South Korea before and after the COVID-19 pandemic while considering the changes in lifestyle patterns resulting from the pandemic.

To address such gaps, we aimed to determine whether the interaction effects between the COVID-19 pandemic and regional deprivation influenced SRH in South Korea.

## Methods

### Study design and setting

This cross-sectional study used data from the Korea Community Health Survey (KCHS) conducted in 2018, 2019, 2020, and 2021 by the Korea Disease Control and Prevention Agency to confirm the interaction effects between the COVID-19 pandemic and regional deprivation on SRH. The KCHS is conducted annually from August 16 to October 31 by public health centers nationwide and targets adults aged 19 years and older. Trained surveyors visit sample households selected using stratified cluster sampling and conduct one-on-one interviews (or electronic surveys) with the final sample households. The survey comprises household and individual components. The household component includes variables such as household type and household income. The individual components include variables related to health behaviors, medical service utilization, prevalent diseases, vaccination, accidents and poisoning, activity limitations and quality of life, healthcare facility utilization, education, employment status, women’s health, cardiopulmonary resuscitation, and socio-physical environmental factors [18–21].

In addition to the KCHS data, we used data from the Population and Housing Census. This is a basic statistical survey conducted by the government to determine the size and characteristics of the South Korean population and their housing. Although statistics on population, households, and housing based on administrative data using the registration census are produced annually, a field survey is conducted every five years to collect the practical data needed for policymaking on welfare, economy, transportation, and so on in each region; finally, a 20% sample of all households in South Korea is selected for the field survey. As this study used publicly available data that lacked personal identifiers, institutional review board or ethics committee approval was not sought.

### Participants

**Fig. 1:**
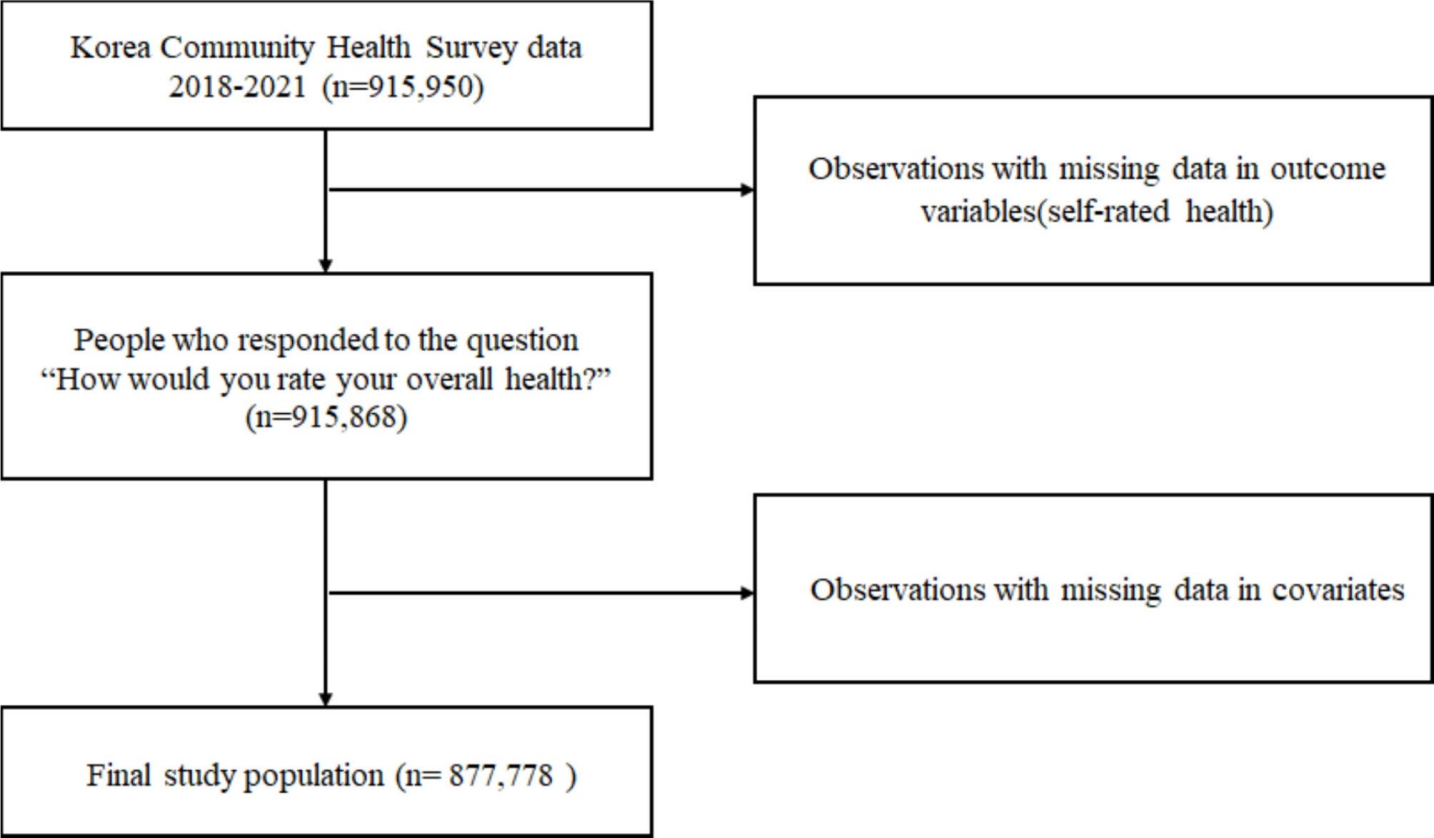
Selection process of the study population

The participants were South Koreans aged 19 years or older living in 17 cities and counties in South Korea, who participated in the 2018–2021 KCHS. A total of 915,950 adults (228,340 in 2018; 229,099 in 2019; 229,269 in 2020; 229,242 in 2021) completed the survey. A total of 38,172 observations (approximately 4%) with missing data on the outcome variable were excluded from the analyses. A total of 877,778 eligible participants (214,929 in 2018; 219,938 in 2019; 219,907 in 2020; 223,004 in 2021) were included in the analysis (Fig. 1).

### Variables

#### Dependent variable

The dependent variable in this study, SRH, consists of a single item. This measurement method is one of the most widely employed approaches to assess general health status in health research. It is relatively simple to measure and allows for international comparisons. SRH was assessed using the question, “How would you rate your overall health?” with the following response options: “very good,” “good,” “fair,” “poor,” and “very poor.” Based on previous studies, we classified (1) individuals who responded “very good” or “good” as the “high” group and (2) individuals who responded “fair,” “poor,” or “very poor” as the “low” group. Participants who responded with “refusal” or “don’t know” were not included in the analysis.

#### Variable of interest

The variable of interest was the interaction between the COVID-19 pandemic (pre-COVID-19/post-COVID-19) and regional socioeconomic level. As the first case of COVID-19 in South Korea was diagnosed on January 20, 2020, the years 2018 and 2019 were classified as pre-COVID-19, whereas 2020 and 2021 were classified as post-COVID-19 [22]. The socioeconomic status of the region was measured using the neighborhood deprivation index. This index extends the traditional concept of poverty, which is defined in terms of resource deprivation or material needs, by including non-monetary resources such as capabilities and social participation to measure multidimensional deprivation in a community [23]. The level of community deprivation was categorized into below-average (advantaged) and above-average (disadvantaged) groups based on the national average neighborhood deprivation index.

We used data from the 2015 Population and Housing Census to calculate the neighborhood deprivation index. The index was calculated based on nine indicators (low social class, deteriorated housing environment, low educational level, car non-ownership, single-person households, divorced or separated status, female-headed households, older population, and non-residence in apartments), and standardized z-scores were calculated for each indicator. The z-scores were then summed to obtain the overall index. This index was applied at the administrative district level [23].

The interaction variable between the COVID-19 pandemic and regional socioeconomic level (COVID-19–neighborhood deprivation) was categorized into four groups based on the COVID-19 pandemic status and regional socioeconomic level. The categories are as follows: pre-COVID-19–advantaged (“pre in advantaged”), pre-COVID-19–disadvantaged (“pre in disadvantaged”), post-COVID-19–advantaged (“post in advantaged”), and post-COVID-19–disadvantaged (“post in disadvantaged”) [24, 25].

#### Covariates

Other covariates were considered, including the participants’ sociodemographic status (gender, age, income level, and employment status) and other related factors that could affect SRH, such as perceived stress, experiences of depressive symptoms, alcohol consumption, smoking status, physical activity, experiences of hypertension, and experiences of diabetes.

### Statistical analysis

Descriptive statistics of SRH were presented based on the participants’ demographic and socioeconomic characteristics. Chi-square tests were conducted to examine differences in SRH based on the participants’ characteristics, presence of the COVID-19 pandemic, and socioeconomic status of participants’ residential areas. Multivariate regression analysis was employed to determine the relationship between SRH and the interaction of the COVID-19 pandemic status with the socioeconomic level of residential areas. All statistical analyses were performed using SAS version 9.4 (SAS Institute Inc., Cary, NC, USA).

## Results

Table 1 presents the general characteristics of the participants. Of the 877,778 participants, 39.6% (*n* = 347,469) exhibited high SRH. The proportion of individuals who rated their health as high varied depending on the interaction between the COVID-19 pandemic and the socioeconomic level of their residential areas. Specifically, in the “pre in disadvantaged” group, the proportion of individuals with high SRH was the lowest at 31.4% (*n* = 61,588). In the “post in advantaged” group, the proportion of individuals with high SRH was the highest at 47.7% (*n* = 116,143).

**Table 1:** Characteristics of the study participants

Variable	Self-Rated Health (SRH)						
	Total		High		Low		
	*N*	%	*N*	%	*N*	%	*p*-value
**Total**	877,778	100.0	347,469	39.6	530,309	60.4	
**Covid-19-Neighborhood deprivation**							
Pre in disadvantaged	195,986	22.3	61,588	31.4	134,398	68.6	< 0.0001
Pre in advantaged	238,881	27.2	89,123	37.3	149,758	62.7	
Post in disadvantaged	199,462	22.7	80,615	40.4	118,847	59.6	
Post in advantaged	243,449	27.7	116,143	47.7	127,306	52.3	< 0.0001
**Gender**							
Women	482,095	54.9	169,291	35.1	312,804	64.9	< 0.0001
Men	395,683	45.1	178,178	45.0	217,505	55.0	
**Age group**							
≥ 70	199,856	22.77	45,140	22.6	154,716	77.4	< 0.0001
60–69	170,768	19.45	58,506	29.3	112,262	56.2	
50–59	169,359	19.29	69,237	34.6	100,122	50.1	
40–49	139,922	15.9	62,706	44.8	77,216	55.2	
30–39	103,197	11.8	52,777	51.1	50,420	48.9	
18–29	94,676	10.8	59,103	62.4	35,573	37.6	
**Income level (quartiles)**							
Q1 (lowest)	211,910	24.1	51,070	24.1	160,840	75.9	< 0.0001
Q2	195,874	22.3	73,351	37.4	122,523	62.6	
Q3	220,187	25.1	97,945	44.5	122,242	55.5	
Q4 (highest)	249,807	28.5	125,103	50.1	124,704	49.9	
**Employment status**		0.0					
Unemployed	333,599	38.0	106,766	32.0	226,833	68.0	< 0.0001
Currently employed	544,179	62.0	240,703	44.2	303,476	55.8	
**Perceived stress**		0.0					
Much	197,116	22.5	58,702	29.8	138,414	70.2	< 0.0001
Less	680,662	77.5	288,767	42.4	391,895	57.6	
**Experiences of depression**		0.0					
No	824,035	93.9	336,166	40.8	487,869	59.2	< 0.0001
Yes	53,743	6.1	11,303	21.0	42,440	79.0	
**Alcohol use**		0.0					
Yes	696,109	79.3	288,845	41.5	407,264	58.5	< 0.0001
No	181,669	20.7	58,624	32.3	123,045	67.7	
**Smoking**		0.0					
Current smoker	145,443	16.6	61,189	42.1	84,254	57.9	< 0.0001
Ex-smoker	165,354	18.8	65,211	39.4	100,143	60.6	
Non-smoker	566,981	64.6	221,069	39.0	345,912	61.0	
**Physical activity**		0.0					
No	515,241	58.7	186,888	36.3	328,353	63.7	< 0.0001
Yes	362,537	41.3	160,581	44.3	201,956	55.7	
**Experiences of Hypertension**							
Yes	248,157	28.3	59,818	24.1	188,339	75.9	< 0.0001
No	629,621	71.7	287,651	45.7	341,970	54.3	
**Experiences of Diabetes**							
Yes	101,728	11.6	18,989	18.7	82,739	81.3	< 0.0001
No	776,050	88.4	328,480	42.3	447,570	57.7	

Table 2 presents the logistic regression results regarding factors related to SRH, with a focus on the effects of COVID-19 and neighborhood deprivation. Compared with the “pre in disadvantaged” group, the “pre in advantaged” group exhibited lower odds of reporting a favorable SRH (0.96 [95% CI 0.94–0.98; *P* < .0001]). Meanwhile, the “post in disadvantaged” group (2.25 [95% CI 2.17–2.34; *P* < .0001]) and “post in advantaged” group (2.29 [95% CI 2.21–2.38; *P* < .0001]) showed significantly higher odds of reporting a favorable SRH compared with the “pre in disadvantaged” group.

**Table 2:** Factors related with self-rated health by COVID-19-neighborhood deprivation

Variable	Self-Rated Health (SRH)			
	OR	95% CI		*p*-value
**Covid-19-Neighborhood deprivation**				
Pre in disadvantaged	1.00			
Pre in advantaged	0.96	0.94	0.98	< 0.0001
Post in disadvantaged	2.25	2.17	2.34	< 0.0001
Post in advantaged	2.29	2.21	2.38	< 0.0001
**Gender**				
Women	1.00			
Men	1.59	1.57	1.62	< 0.0001
**Age group**				
70+	1.00			
60–69	1.30	1.27	1.33	< 0.0001
50–59	1.33	1.30	1.37	< 0.0001
40–49	1.40	1.36	1.44	< 0.0001
30–39	1.83	1.78	1.88	< 0.0001
18–29	2.69	2.61	2.76	< 0.0001
**Income level (quartiles)**				
Q1 (lowest)	1.00			
Q2	1.25	1.22	1.28	< 0.0001
Q3	1.40	1.37	1.43	< 0.0001
Q4 (highest)	1.62	1.59	1.66	< 0.0001
**Employment status**				
Unemployed	1.00			
Currently employed	1.21	1.20	1.23	< 0.0001
**Perceived stress**				
Much	1.00			
Less	1.95	1.92	1.98	< 0.0001
**Experiences of depression**				
Yes	1.00			
No	1.83	1.77	1.88	< 0.0001
**Alcohol use**				
Yes	1.00			
No	1.01	0.99	1.03	0.3552
**Smoking**				
Current smoker	1.00			
Ex-smoker	1.15	1.13	1.18	0.0083
Non-smoker	1.39	1.36	1.42	< 0.0001
**Physical activity**				
No	1.00			
Yes	1.33	1.31	1.35	< 0.0001
**Experiences of Hypertension**				
Yes	1.00			
No	1.71	1.68	1.74	< 0.0001
**Experiences of Diabetes**				
Yes	1.00			
No	2.21	2.16	2.27	< 0.0001

* Values are presented as number (%) or mean ± standard deviation

These findings indicated a notable increase in the ORs for reporting a favorable SRH in the post-COVID-19 period. However, there were marginal differences in ORs based on the regional socioeconomic level, suggesting minimal disparities in SRH with respect to regional socioeconomic factors.

To evaluate the additional risk posed by the interaction between COVID-19 and regional deprivation on SRH, we conducted further analyses presented in Appendix 2. These analyses applied measures such as the Relative Excess Risk due to Interaction (RERI), Attributable Proportion due to Interaction (AP), and Synergy Index (SI). The RERI value of 0.04 (95% CI: 0.04–0.05) suggested an additional risk when both factors were present. The AP value of 0.03 (95% CI: 0.02–0.03) represented the proportion of risk attributable to the interaction, while the SI value of 1.07 (95% CI: 1.07–1.08) indicated a positive interaction between the two factors.

## Discussion

We analyzed SRH in relation to the occurrence of the COVID-19 pandemic and regional socioeconomic level. By comparing the average SRH before and after the COVID-19 pandemic, with the “pre in disadvantaged” group as the reference, we observed increased odds of reporting high SRH after the COVID-19 pandemic. However, we found minimal or inconclusive differences in SRH based on the regional socioeconomic level. This result suggests that the impact of the COVID-19 pandemic may have overshadow any regional differences in socioeconomic levels on SRH.

Additionally, we conducted subgroup analyses according to age and income levels to further explore these relationships. As shown in Appendix 3, for individuals aged ≥ 60 years, the OR for reporting high SRH was 1.15 (95% CI 1.11–1.19; *P* < .0001) in the “pre in advantaged” group, 2.44 (95% CI 2.31–2.59; *P* < .0001) in the “post in disadvantaged” group, and 2.77 (95% CI 2.62–2.93; *P* < .0001) in the “post in advantaged” group, relative to the “pre in disadvantaged” group. This indicates that the impact of regional deprivation on SRH significantly varies across different age groups, with older adults showing more pronounced differences.

Similarly, among individuals in the lowest income quartile, the OR for reporting high SRH was 1.10 (95% CI 1.05–1.15; *P* < .0001) in the “pre in advantaged” group, 2.65 (95% CI 2.45–2.87; *P* < .0001) in the “post in disadvantaged” group, and 3.01 (95% CI 2.78–3.26; *P* < .0001) in the “post in advantaged” group, relative to the “pre in disadvantaged” group (Appendix 4). This indicates that income level influences the relationship between regional deprivation and SRH, with the lowest income group showing the most substantial differences. These findings suggest that the health-related variables we adjusted for critically influence the relationship between regional deprivation and SRH. Therefore, it is essential to consider these variables in order to elucidate the nuanced effects of regional deprivation on SRH. Moreover, we conducted additional analyses with different sets of covariates to understand their effects, with the results of these analyses being presented in Appendix 5.

Additionally, we conducted further analyses to examine the interaction effect between the COVID-19 pandemic and neighborhood deprivation on SRH (Appendix 2). The findings indicated that the combined effect of the COVID-19 pandemic and neighborhood deprivation on SRH is greater than the sum of their individual effects. This highlights the importance of considering interaction effects in understanding health disparities during the COVID-19 pandemic.

Individuals’ positive health ratings during the pandemic have various explanations. First, individuals’ health perceptions could have changed because of social comparisons, as people tend to evaluate their health by comparing themselves with others [26]. The survey conducted in this study targeted individuals who had not contracted COVID-19. Individuals who had not been infected with the virus may have evaluated themselves more positively than they would under normal circumstances.

Furthermore, previous studies involving individuals who had not contracted COVID-19 have shown relatively high rates of improved SRH after the pandemic rather than a decline in SRH [7, 14, 15]. A study in France described this finding as the “Eye of the Hurricane” paradox, suggesting that individuals who had not been infected with COVID-19 may have assessed their health more positively than they typically would [15]. In a study with Dutch respondents, the majority of the sample (66.7%) reported the same SRH before and during the pandemic, whereas 10.8% reported a decrease and 22.5% reported an increase [7]. A similar result was found in a study by Peters, in which variations in SRH before and during the pandemic were studied using a large German sample [14]. More than half of the participants (56%) stated that their SRH had not changed, 32% said it had improved, and 12% said it had decreased. This result aligns with the findings of the present study, which revealed higher odds of positive SRH after the COVID-19 pandemic.

Second, the robust implementation of effective containment measures in South Korea may have influenced individuals’ SRH. South Korea received substantial recognition for its successful efforts to control the spread of COVID-19 during the height of the pandemic. Among the 33 member countries of the Organisation for Economic Cooperation and Development, South Korea was evaluated as the top performer in COVID-19 containment [27]. The country efficiently carried out prompt testing, contact tracing, and isolation of confirmed cases, while the majority of the population adhered to mask wearing, resulting in minimal economic impact of the pandemic [27]. Against this backdrop, the social comparison mechanism may come into play and influence individuals’ SRH. Effective public health responses can alleviate anxiety and stress, leading to improved overall well-being. For example, a previous study reported a correlation of effective COVID-19 precautionary measures with reduced psychological distress and improved mental health outcomes within the general population [28]. Given the efficient implementation of disease prevention measures in South Korea, [29] the observed differences between before and after the COVID-19 pandemic may have had a more substantial effect on SRH than variations between neighborhood deprivation.

This study has certain limitations. First, it did not include objective disease indicators, meaning that the observed increase in average SRH may not reflect an actual improvement in objective health. When comparing the number of individuals with one or more chronic illnesses for each year, we observed an overall increasing trend: 32.3% in 2018, 32.7% in 2019, 32.1% in 2020, and 33.4% in 2021. These findings suggest that the prevalence of chronic conditions among individuals did not decline during the study period (Table 3). Future research should include objective health indicators to provide a more comprehensive assessment of health trends.

**Table 3:** Number of individuals with chronic diseases by year

	Total										p-value
			2018		2019		2020		2021		
	N	%	N	%	N	%	N	%	N	%	
HTN or DM	877,778	100.0	214,929	24.5	219,938	25.1	219,907	25.1	223,004	25.4	
No	591,371	67.4	145,602	67.7	148,055	67.3	149,246	67.9	148,468	66.6	< 0.0001
Yes	286,407	**32.6**	69,327	**32.3**	71,883	**32.7**	70,661	**32.1**	74,536	**33.4**	

*Values are presented as number (%) or mean ± standard deviation

Second, we conducted a cross-sectional study because of the limitations of the data; the data used were not followed up, and interviewers were recruited every year. Nevertheless, we exerted efforts to minimize these limitations by using reliable data that could represent the population of South Korea. Additionally, we provided detailed demographic characteristics of the study participants for each year in Appendix 6, which showed stability across the years, and thus reinforces the robustness of our analysis despite the cross-sectional design.

Third, the international comparisons of SRH have limitations. Differences in survey question construction, especially, differences in survey scales, can affect the comparability of responses [30]. The question-and-answer categories used in the survey questions vary from one country to another, thus limiting international comparisons. Internationally standardized indicators should be developed to address this limitation.

Nevertheless, the strength of the current study relative to existing research is that it analyzed SRH before and after COVID-19, in combination with the neighborhood effect. Moreover, this study is the first of its kind in the context of South Korea. While accurate international comparisons are not possible, this study can be used to compare trends in other international studies that have analyzed subjective health before and after COVID-19.

In conclusion, the SRH status showed an overall increase in the “post in disadvantaged” and “post in advantaged” groups relative to the “pre in disadvantaged” group. The possible reasons for this difference include changes in individuals’ health perceptions through social comparisons (e.g., “Eye of the Hurricane”) and the effective implementation of containment measures in South Korea.

## Conclusions

The study conclusively demonstrates an overall improvement in SRH in both “post in disadvantaged” and “post in advantaged” groups compared to the “pre in disadvantaged” group, after the onset of the COVID-19 pandemic. This unexpected improvement suggests a significant impact of the pandemic on individuals’ perception of their health, potentially influenced by social comparison phenomena, such as the “Eye of the Hurricane” effect, and the successful implementation of COVID-19 containment measures in South Korea. These findings underscore the complex interplay between a public health crisis and social factors affecting health perceptions, highlighting the need for continued exploration of these dynamics to inform public health strategies and interventions.

### Electronic supplementary material

Below is the link to the electronic supplementary material.

Supplementary Material 1

## Data Availability

This study comprised data from the Korea Community Health Survey conducted in 2018, 2019, 2020, and 2021 by the Korea Disease Control and Prevention Agency. Data can be downloaded from the KCHS official website (https://chs.kdca.go.kr/chs/index.do).

## Abbreviations

AP: Attributable proportion due to interaction
COVID-19: Coronavirus disease 2019
KCHS: Korea community health survey
SRH: Self-rated health
RERI: Relative Excess risk due to interaction
SI: Synergy index
