# Supplementary material for: Interaction effects of the COVID-19 pandemic and regional deprivation on self-rated health: a cross-sectional study
Source: BMC Public Health. 2024 Sep 2;24:2382. doi: 10.1186/s12889-024-19814-x (PMC11367910; doi:10.1186/s12889-024-19814-x)
Supplement: Supplementary file 1 — Supplementary Material 1 [file 12889_2024_19814_MOESM1_ESM.pdf]

Appendix1. Timeline of COVID-19 measures in South Korea

Date	Event/Measure
20-Jan-2020	First COVID-19 case reported in South Korea.
23-Feb-2020	Alert level raised to the highest (Red); extensive contact tracing initiated.
29-Feb-2020	Daily new cases peak at 909; extensive testing ramped up.
22-Mar-2020	Social distancing guidelines introduced nationwide.
06-May-2020	Transition to "everyday life quarantine"; eased social distancing.
15-Aug-2020	Resurgence of cases; stricter social distancing measures reinstated.
30-Aug-2020	Level 2.5 social distancing measures in the Seoul metropolitan area.
24-Nov-2020	Stricter distancing (Level 2) in greater Seoul due to rising cases.
08-Dec-2020	Nationwide social distancing raised to Level 2.5.
24-Dec-2020	Year-end gatherings restricted, including Christmas and New Year.
04-Jan-2021	Social distancing measures extended due to continued high case numbers.
26-Feb-2021	Vaccination campaign begins with healthcare workers and high-risk groups.
12-Jul-2021	Social distancing Level 4 implemented in Seoul and surrounding areas.
23-Aug-2021	Level 4 social distancing extended in the Seoul metropolitan area.
03-Sep-2021	Nationwide measures adjusted; Level 3 applied in non-metropolitan areas.
01-Nov-2021	"Living with COVID-19" plan starts; gradual easing of restrictions.
18-Dec-2021	Social distancing measures tightened again due to Omicron variant concerns.

Appendix2. Additive interaction analysis between COVID-19 and neighborhood deprivation for Self-rated Health

	Disadvantaged area	Advantaged area			
	OR(95%CI)		RERI(95%CI)	AP(95%CI)	SI(95%CI)
**Pre-Covid19**	1.00 (Ref.)	1.04 (1.02-1.07)	0.04 (0.04-0.05)	0.03 (0.02-0.03)	1.07 (1.07-1.08)
**Post-Covid19**	0.44 (0.43-0.46)	0.44 (0.42-0.45)			

* RERI the relative excess risk due to interaction, AP the attributable proportion due to interaction, SI the synergy index.

** Models is adjusted by gender, age, income level, employment status, experiences of depression, perceived stress, alcohol consumption, smoking, physical activity, experiences of Hypertension, experiences of Diabetes.

Appendix3. Results of factors related with Self-rated health by COVID-19-neighborhood deprivation by age

Variable	18-39 (N=197,873)				40-59 (N=309,281)				60+ (N=370,624)			
	OR	95% CI		p-value	OR	95% CI		p-value	OR	95% CI		p-value
**Covid-19-Neighborhood deprivation**												
Pre in disadvantaged	1.00				1.00				1.00			
Pre in advantaged	0.87	0.84	0.91	<.0001	0.93	0.90	0.96	<.0001	1.15	1.11	1.19	<.0001
Post in disadvantaged	2.18	2.03	2.34	<.0001	2.21	2.09	2.34	<.0001	2.44	2.31	2.59	<.0001
Post in advantaged	2.10	1.98	2.24	<.0001	2.17	2.06	2.29	<.0001	2.77	2.62	2.93	<.0001
**Gender**												
Women	1.00				1.00				1.00			
Men	1.58	1.54	1.63	<.0001	1.49	1.44	1.53	<.0001	1.76	1.70	1.82	<.0001
**Income level (quartiles)**												
Q1 (lowest)	1.00				1.00				1.00			
Q2	1.05	0.99	1.11	<.0001	1.22	1.17	1.28	<.0001	1.34	1.30	1.38	0.0002
Q3	1.10	1.05	1.17	0.2382	1.38	1.32	1.44	<.0001	1.58	1.53	1.63	<.0001
Q4 (highest)	1.35	1.28	1.42	<.0001	1.61	1.54	1.68	<.0001	1.74	1.68	1.80	<.0001
**Employment status**												
Unemployed	1.00				1.00				1.00			
Currently employed	0.97	0.95	0.99	0.0163	1.32	1.29	1.36	<.0001	1.50	1.47	1.54	<.0001
**Perceived stress**												
Much	1.00				1.00				1.00			
Less	2.06	2.01	2.12	<.0001	1.79	1.75	1.84	<.0001	2.02	1.95	2.09	<.0001
**Experiences of depression**												
Yes	1.00				1.00				1.00			
No	1.93	1.83	2.03	<.0001	1.72	1.64	1.80	<.0001	1.76	1.66	1.86	<.0001
**Alcohol use**												

Yes	1.00				1.00				1.00			
No	1.19	1.14	1.24	<.0001	1.02	0.99	1.06	0.1309	0.93	0.91	0.96	<.0001
**Smoking**												
Current smoker	1.00				1.00				1.00			
Ex-smoker	1.05	1.00	1.09	<.0001	1.14	1.10	1.17	0.0405	1.07	1.03	1.12	<.0001
Non-smoker	1.38	1.34	1.43	<.0001	1.37	1.33	1.42	<.0001	1.31	1.26	1.37	<.0001
**Physical activity**												
No	1.00				1.00				1.00			
Yes	1.34	1.31	1.38	<.0001	1.29	1.27	1.32	<.0001	1.47	1.44	1.50	<.0001
**Experiences of Hypertension**												
Yes	1.00				1.00				1.00			
No	2.69	2.48	2.91	<.0001	1.85	1.80	1.91	<.0001	1.53	1.50	1.57	<.0001
**Experiences of Diabetes**												
Yes	1.00				1.00				1.00			
No	3.66	3.15	4.25	<.0001	2.77	2.65	2.90	<.0001	1.89	1.84	1.95	<.0001

* Values are presented as odds ratio (95% confidence interval)

Appendix4. Results of factors related with Self-rated health by COVID-19-neighborhood deprivation by income

Variable	Q1 (N= 211,910)				Q2 (N=195,874)				Q3 (N=220,187)				Q4 (N=249,807)			
	OR	95% CI		p-value	OR	95% CI		p-value	OR	95% CI		p-value	OR	95% CI		p-value
**Covid-19-Neighborhood deprivation**																
Pre in disadvantaged	1.00				1.00				1.00				1.00			
Pre in advantaged	1.10	1.05	1.15	<.0001	1.00	0.96	1.04	<.0001	0.89	0.86	0.93	<.0001	0.93	0.89	0.96	<.0001
Post in disadvantaged	2.65	2.45	2.87	<.0001	2.48	2.31	2.66	<.0001	2.09	1.96	2.24	<.0001	2.13	1.99	2.29	<.0001
Post in advantaged	3.01	2.78	3.26	<.0001	2.52	2.35	2.70	<.0001	2.07	1.95	2.21	<.0001	2.11	1.99	2.25	<.0001
**Gender**																
Women	1.00				1.00				1.00				1.00			
Men	1.62	1.55	1.70	<.0001	1.54	1.48	1.60	<.0001	1.62	1.57	1.67	<.0001	1.61	1.56	1.65	<.0001
**Age group**																
70+	1.00				1.00				1.00				1.00			
60-69	1.19	1.14	1.24	<.0001	1.17	1.12	1.22	<.0001	1.34	1.27	1.41	<.0001	1.54	1.44	1.65	<.0001
50-59	1.25	1.18	1.32	<.0001	1.21	1.15	1.27	<.0001	1.29	1.22	1.37	<.0001	1.54	1.44	1.64	<.0001
40-49	1.57	1.46	1.68	0.2896	1.29	1.22	1.36	<.0001	1.39	1.31	1.47	<.0001	1.54	1.44	1.65	<.0001
30-39	2.15	1.97	2.35	<.0001	1.75	1.65	1.84	<.0001	1.80	1.70	1.91	<.0001	1.98	1.85	2.11	<.0001
18-29	3.57	3.33	3.83	<.0001	2.46	2.33	2.60	<.0001	2.51	2.36	2.66	<.0001	2.90	2.72	3.10	<.0001
**Employment status**																
Unemployed	1.00				1.00				1.00				1.00			
Currently employed	1.48	1.43	1.53	<.0001	1.30	1.26	1.34	<.0001	1.14	1.11	1.17	<.0001	1.15	1.12	1.18	<.0001
**Perceived stress**																
Much	1.00				1.00				1.00				1.00			
Less	2.15	2.05	2.25	<.0001	1.95	1.89	2.02	<.0001	1.89	1.83	1.94	<.0001	1.93	1.88	1.98	<.0001
**Experiences of depression**																
Yes	1.00				1.00				1.00				1.00			
No	1.90	1.78	2.04	<.0001	1.78	1.68	1.90	<.0001	1.75	1.65	1.86	<.0001	1.86	1.76	1.96	<.0001

**Alcohol use**																
Yes	1.00				1.00				1.00				1.00			
No	0.96	0.93	1.00	0.0384	1.01	0.97	1.04	0.7365	1.03	0.99	1.07	0.1197	1.04	1.01	1.08	0.0232
**Smoking**																
Current smoker	1.00				1.00				1.00				1.00			
Ex-smoker	1.12	1.06	1.18	0.0953	1.15	1.10	1.20	0.5991	1.17	1.13	1.21	0.6082	1.14	1.10	1.19	0.0114
Non-smoker	1.35	1.27	1.43	<.0001	1.34	1.28	1.40	<.0001	1.39	1.34	1.44	<.0001	1.42	1.37	1.47	<.0001
**Physical activity**																
No	1.00				1.00				1.00				1.00			
Yes	1.43	1.38	1.47	<.0001	1.34	1.31	1.38	<.0001	1.32	1.29	1.35	<.0001	1.30	1.27	1.33	<.0001
**Experiences of Hypertension**																
Yes	1.00				1.00				1.00				1.00			
No	1.57	1.52	1.63	<.0001	1.63	1.57	1.69	<.0001	1.73	1.67	1.79	<.0001	1.83	1.77	1.89	<.0001
**Experiences of Diabetes**																
Yes	1.00				1.00				1.00				1.00			
No	1.84	1.75	1.93	<.0001	2.22	2.12	2.33	<.0001	2.20	2.09	2.31	<.0001	2.57	2.44	2.71	<.0001

* Values are presented as odds ratio (95% confidence interval)

Appendix5. Adjusted Odds Ratios for Self-rated health by COVID-19 and regional deprivation with varying covariates

Variable	Model 1*				Model 2**				Model 3***			
	OR	95% CI		p-value	OR	95% CI		p-value	OR	95% CI		p-value
**Covid-19-Neighborhood deprivation**												
Pre in disadvantaged	1.00				1.00				1.00			
Pre in advantaged	1.12	1.10	1.14	<.0001	1.11	1.08	1.13	<.0001	1.09	1.07	1.11	<.0001
Post in disadvantaged	2.03	1.95	2.34	<.0001	2.07	1.99	2.15	<.0001	2.16	2.08	2.25	<.0001
Post in advantaged	2.38	2.29	2.38	<.0001	2.40	2.31	2.48	<.0001	2.49	2.40	2.58	<.0001

* Models1 is adjusted by gender.

**Models2 is adjusted by gender, employment status, alcohol consumption, smoking.

***Models3 is adjusted by gender, employment status, alcohol consumption, smoking, perceived stress, physical activity, experiences of Hypertension, experiences of Diabetes.

Appendix6. General characteristics by years

Variable	Years							
	2018		2019		2020		2021	
	N	%	N	%	N	%	N	%
**Total**	214,929	100.0	219,938	100.0	219,907	100.0	223,004	100.0
**Neighborhood deprivation**								
Disadvantaged area	96,579	44.94	99,407	45.20	99,284	45.15	100,178	44.92
Advantaged area	118,350	55.06	120,531	54.80	120,623	54.85	122,826	55.08
**Gender**								
Women	118,774	55.26	121,556	55.27	120,319	54.71	121,446	54.46
Men	96,155	44.74	98,382	44.73	99,588	45.29	101,558	45.54
**Age group**								
70+	48,780	22.70	51,038	23.21	49,486	22.50	50,552	22.67
60-69	39,663	18.45	42,629	19.38	42,665	19.40	45,811	20.54
50-59	41,853	19.47	42,534	19.34	42,882	19.50	42,090	18.87
40-49	35,158	16.36	34,989	15.91	34,871	15.86	34,904	15.65
30-39	27,156	12.63	26,046	11.84	24,641	11.21	25,354	11.37
18-29	22,319	10.38	22,702	10.32	25,362	11.53	24,293	10.89
**Income level (quartiles)**								
Q1 (lowest)	53,144	24.73	52,006	23.65	53,899	24.51	52,861	23.70
Q2	48,033	22.35	48,617	22.10	49,801	22.65	49,423	22.16
Q3	55,561	25.85	56,365	25.63	54,413	24.74	53,848	24.15
Q4 (highest)	58,191	27.07	62,950	28.62	61,794	28.10	53,848	24.15
**Employment status**								
Unemployed	81,936	38.12	82,900	37.69	85,847	39.04	82,916	37.18
Currently employed	132,993	61.88	137,038	62.31	134,060	60.96	140,088	62.82
**Perceived stress**								

Much	50,188	23.35	48,610	22.10	48,717	22.15	49,601	22.24
Less	164,741	76.65	171,328	77.90	171,190	77.85	173,403	77.76
**Experiences of depression**								
No	12,239	5.69	13,440	6.11	12,246	5.57	15,818	7.09
Yes	202,690	94.31	206,498	93.89	207,661	94.43	207,186	92.91
**Alcohol use**								
Yes	178,763	83.17	177,879	80.88	166,627	75.77	172,840	77.51
No	36,166	16.83	42,059	19.12	53,280	24.23	50,164	22.49
**Smoking**								
Current smoker	37,211	17.31	36,466	16.58	35,916	16.33	35,850	16.08
Ex-smoker	38,993	18.14	44,229	20.11	40,010	18.19	42,122	18.89
Non-smoker	138,725	64.54	139,243	63.31	143,981	65.47	145,032	65.04
**Physical activity**								
No	119,622	55.66	131,496	59.79	133,718	60.81	130,405	58.48
Yes	95,307	44.34	88,442	40.21	86,189	39.19	92,599	41.52
**Experiences of Hypertension**								
Yes	60,843	28.31	62,369	28.36	60,882	27.69	64,063	28.73
No	154,086	71.69	157,569	71.64	159,025	72.31	158,941	71.27
**Experiences of Diabetes**								
Yes	23,693	11.02	25,154	11.44	25,455	11.58	27,426	12.30
No	191,236	88.98	194,784	88.56	194,452	88.42	195,578	87.70
